# Effect of Green Tea Extract and Soy Isoflavones on the Pharmacokinetics of Rosuvastatin in Healthy Volunteers

**DOI:** 10.3389/fnut.2022.850318

**Published:** 2022-03-24

**Authors:** Weiwei Zeng, Miao Hu, Hon Kit Lee, Elaine Wat, Clara Bik San Lau, Chung Shun Ho, Chun Kwok Wong, Brian Tomlinson

**Affiliations:** ^1^Shenzhen Baoan Women's and Children's Hospital, Jinan University, Shenzhen, China; ^2^Department of Medicine & Therapeutics, The Chinese University of Hong Kong, Prince of Wales Hospital, Hong Kong, Hong Kong SAR, China; ^3^Department of Chemical Pathology, The Chinese University of Hong Kong, Hong Kong, Hong Kong SAR, China; ^4^Department of Clinical Pathology, Tuen Mun Hospital, Hong Kong, Hong Kong SAR, China; ^5^Institute of Chinese Medicine and State Key Laboratory of Research on Bioactivities and Clinical Applications of Medicinal Plants, The Chinese University of Hong Kong, Hong Kong, Hong Kong SAR, China; ^6^Faculty of Medicine, Macau University of Science and Technology, Macau, China

**Keywords:** ABCG2, drug interaction, EGCG, green tea, rosuvastatin, soy isoflavones

## Abstract

**Background and Aim:**

Green tea and soy products are extensively consumed in daily life. Research has shown that green tea catechins and soy isoflavones may influence the activity of drug metabolizing enzymes and drug transporters. We examined whether regular consumption of green tea extract or soy isoflavones affected the pharmacokinetics of a single dose of rosuvastatin in healthy subjects and whether any interactions were influenced by the polymorphism in the drug transporter ABCG2.

**Study Design:**

This was an open-label, three-phase randomized crossover study with single doses of rosuvastatin.

**Methods:**

Healthy Chinese male subjects were given a single dose of rosuvastatin 10 mg on 3 occasions: 1. without herbs; 2. with green tea extract; 3. with soy isoflavone extract. The green tea and soy isoflavone extract were given at a dose containing EGCG 800 mg once daily or soy isoflavones−80 mg once daily for 14 days before statin dosing and at the same time as the statin dosing with at least 4-weeks washout period between phases.

**Results:**

Twenty healthy male subjects completed the study and the intake of green tea extract significantly reduced the systemic exposure to rosuvastatin by about 20% reducing AUC_0−24*h*_ from [geometric mean (% coefficient of variation)] 108.7 (28.9) h·μg/L to 74.1 (35.3) h·μg/L and C_max_ from 13.1 (32.2) μg/L to 7.9 (38.3) μg/L (*P* < 0.001 for both), without affecting the elimination half-life. The ABCG2 421C>A polymorphism had a significant effect on rosuvastatin exposure but no impact on the interaction with green tea. Soy isoflavones had no significant effect on rosuvastatin pharmacokinetics.

**Conclusion:**

This study showed that repeated administration of green tea extract significantly reduced the systemic exposure of rosuvastatin in healthy volunteers. These effects might be predicted to either reduce or increase the lipid-lowering effect of rosuvastatin depending on the mechanism of the effect.

## Introduction

Certain food and beverage components, such as flavonoids, are thought to have health benefits, particularly in preventing cardiovascular diseases (1). These foods and herbal medicines are widely used by patients with cardiovascular diseases and interactions between these and cardiovascular drugs have been frequently reported (2, 3). Interactions may have beneficial effects or, more often, adverse reactions such as toxicity or treatment failure and may be influenced by multiple environmental and/or genetic factors (4, 5). Herbal medicines and food components usually affect the pharmacokinetics of concomitant drugs by inhibition or induction of cytochrome P450 (CYP) enzymes or drug transporters (6).

The HMG-CoA reductase inhibitors or statins are first-line treatments for hypercholesterolaemia and for preventing cardiovascular events. Rhabdomyolysis is a rare but potentially life-threatening side effect of the statins, but milder degrees of myopathy and myalgia are more common (7–9). The exact mechanism of statin myotoxicity is uncertain, but it is usually associated with higher plasma concentrations of the drug due to variations in drug metabolism or drug-drug interactions (10–12). Drug transporters play an important role in the disposition, safety and efficacy of statins (8, 13). Some polymorphisms in the transporter genes have been shown to influence the pharmacokinetics, safety and lipid-lowering effect of certain substrate statins (14–17). In a previous study, we found that the breast cancer resistance protein (BCRP) or ATP binding cassette (ABC) G2 421C>A polymorphism played a major role in the low-density lipoprotein cholesterol (LDL-C) response to rosuvastatin in Chinese subjects suggesting this transporter is important in the disposition of rosuvastatin (16).

Rosuvastatin is also a substrate for other transporters including hepatic organic anion transporting polypeptide 1B1 (OATP1B1, gene SLCO1B1), OATP1B3, multidrug resistance-associated protein 4 (MRP4, gene ABCC4), sodium-taurocholate cotransporting polypeptide (NTCP, gene SLC10A1), intestinal OATP1A2 and OATP2B1 and renal organic anion transporter 3 (OAT3) (18, 19). Certain herbs and foods which interfere with statin metabolism or transport may be associated with an increased risk of myopathy or altered statin efficacy (20, 21).

Flavonoids are polyphenolic compounds widely present in vegetables, fruits and plant derivatives such as soya bean, wine and tea and are also the main components of many herbal health products (1). Studies have shown that flavonoids could inhibit multiple ABC efflux transporters, including P-glycoprotein (P-gp, gene ABCB1), ABCC2 and ABCG2 (22), as well as SLCO1B1 (23).

Whether green tea or soy flavonoids significantly influence transporter activity to alter statin pharmacokinetics and pharmacodynamics is an important topic that needs to be addressed. We chose green tea extract and soy isoflavones as these are widely consumed in daily life. Average green tea consumption in Asian countries is about 3 cups per day, providing 240–320 mg of polyphenols (24). The most abundant polyphenols in green tea are epigallocatechin gallate (EGCG), epigallocatechin (EGC), epicatechin gallate (ECG), and epicatechin (EC) and these are believed to be responsible for the proposed benefits (25). Soybean is another common food eaten boiled or roasted or as soy flour as a food ingredient. Soybeans contain high amounts of isoflavones, some of which are similar to the female hormone estrogen and are considered to be phytoestrogens (26, 27). The average daily intake of soy foods is 54.4 g and 63.6 g for female and male Asian adults, respectively, representing an average of 15–45 mg isoflavones/day (26).

Genetic polymorphisms in drug transporters or enzymes may influence herb-drug interactions substantially, as we reported previously with CYP enzymes (5) and this is important to examine with the statins. In this study, we examined whether green tea extract and soy isoflavones affected the pharmacokinetics of rosuvastatin in healthy subjects and whether these interactions were influenced by the common polymorphism in the ABCG2 transporter.

## Methods

### Subjects

Healthy Chinese male subjects aged 18–45 years were recruited from a pool of over 200 healthy volunteers who had been genotyped for the ABCG2 421C>A (rs2231142) polymorphism. A total of 20 healthy Chinese male subjects were selected for the rosuvastatin pharmacokinetic study based on the ABCG2 421C>A genotypes. The study was reviewed and approved by the Joint Chinese University of Hong Kong-New Territories East Cluster Clinical Research Ethics Committee with reference number CRE-2010.524-T. The study was performed in accordance with the ethical standards laid down in the Declaration of Helsinki and subsequent revisions. Written informed consent was obtained from each participant.

All subjects were required to abstain from any prescription or non-prescription medications 2 weeks before and throughout the study. They were not allowed to take alcohol, tea, grapefruit juice, caffeine, soybean milk or dietary supplements and herbal products 2 weeks before and throughout the entire study period. They were also not allowed to smoke 2 weeks before and throughout the study. Subjects were requested to fast for 10 h before and 4 h after drug administration during the blood sampling sessions. Meals were standardized and consumed at 4 h and 10 h post-dosing. Drinking water was not allowed from 1 h pre-dose to 1 h post-dose except that needed for drug dosing at the time of blood sampling sessions. Subjects were asked to report any adverse effects at each visit to the study center.

### Rosuvastatin-Herb Pharmacokinetic Interaction

This was an open-label, three-phase randomized crossover study. Subjects were given a single dose of rosuvastatin 10 mg (Crestor^®^, Astra Zeneca) on 3 occasions: 1. without herbs; 2. with green tea extract; 3. with soy isoflavones extract. The green tea extract and soy isoflavones extract were given at a dose thought to contain EGCG 800 mg once daily or isoflavones 80 mg once daily for 14 days before rosuvastatin dosing with at least a 4-week washout period between phases. The herbal extracts were present as a powder which was taken in 150 ml water at room temperature. Blood samples were taken at intervals from 0 to 24 h on the rosuvastatin dosing days. During the study, subjects were frequently reminded of the requirements for diet restrictions, and they were requested to record their daily food intake, including the main meals, snacks and beverages of the day by using a simple food diary, which can help to monitor the food compliance during the study.

### Herbal Products

Green tea extract and soy isoflavones extract products were manufactured by the Hong Kong Institute of Biotechnology (HKIB) in accordance with Good Manufacturing Practice (GMP). Standard heavy metal, microbial and pesticides tests were performed to ensure the products fulfilled the safety requirement set out by the Department of Health in Hong Kong. Each green tea extract sachet claimed to contain 800 mg standardized EGCG. Each soy extract sachet claimed to contain 80 mg standardized total isoflavones.

### Quantification of Plasma Concentrations of Rosuvastatin

Plasma concentrations of rosuvastatin were determined by Liquid Chromatography-Tandem Mass Spectrometry (LC-MS/MS) using the corresponding isotopically labeled compound as an internal standard as described (28). The plasma samples were prepared using liquid–liquid extraction with diethyl ether. Chromatographic separation was accomplished on an Xterra MS C18 column (50 × 2.1 mm, 5 μm; Waters, Milford, MA). The mobile phase consisted of a gradient mixture of 0.015 mmol/L ammonium acetate in water (mobile phase A) and methanol (mobile phase B) at a flow rate of 0.4 mL/min. The gradient started at 20% mobile phase B for 0.5 min with a subsequent fast gradient to 100% mobile phase B in 1 min and maintained for another 2.5 min. The gradient was then returned to the initial mobile phase concentration in a chromatographic run of 8 min. MS/MS detection with quantification transition of m/z 480.1–418.1 and qualification transition of m/z 480.1–340.1 was used for rosuvastatin. The lower limit of quantification of rosuvastatin was 0.05 μg/L by using 200 μL plasma. The linear range of the method was from 0.05 to 42.0 μg/L. The coefficients of variation and % deviations from expected values were lower than 14% and within −4.7 to 9.8%, respectively.

### Genotyping

DNA was extracted from the blood samples using High Pure PCR Template Preparation Kits (Roche Applied Science). Subjects were genotyped for the ABCG2 421C>A (rs2231142) polymorphism by using the TaqMan Drug Metabolism Genotyping Assays from Applied Biosystems (Foster City, CA, USA).

### Pharmacokinetic Analysis

The pharmacokinetic parameters of rosuvastatin were calculated using non-compartmental methods with the aid of the computer program WinNolin (version 2.1, Pharsight Corporation). The maximum plasma concentration (C_max_) and time to C_max_ (t_max_) were obtained directly from the observed concentration-time data. The terminal elimination rate constant (λ_Z_) was estimated by linear regression of the terminal portion of the concentration-time curve, and the elimination half-life (*t*_1/2_) was calculated as 0.693/λ_Z_. Systemic exposure to rosuvastatin was evaluated by calculating the AUC using the linear trapezoidal rule and AUC_0−∞_ was calculated as AUC_0−∞_ = AUC_0−t_ + C_t_/K_el_ where C_t_ is the last quantifiable concentration. The oral clearance (CL/F) was calculated as Dose/AUC_0−∞_.

### Statistical Analysis

The primary outcome was the difference in rosuvastatin pharmacokinetic parameters with and without herb consumptions. Logarithmic transformation was used for pharmacokinetic variables, except for t_max_. The pharmacokinetic parameters of rosuvastatin after consumption of green tea or soy isoflavones were compared with those when the drugs were taken without these herbs by repeated measures ANOVA, except for t_max_ values for which the Friedman rank test was used. The geometric mean ratios and 90% confidence intervals (CI) were calculated from the log-transformed values of C_max_ and AUC compared between treatments. The comparisons of the pharmacokinetic parameters and interactions among genotype groups were determined using ANOVA for normally distributed data or the Kruskal-Wallis test for skewed data. Differences were considered statistically significant at *P* < 0.05.

### Sample Size

Significant effects of polymorphisms in drug transporters can be seen for single-dose complete pharmacokinetic studies in small groups of *n* = 6 (29). In a previous study, a herb-drug interaction between baicalin and rosuvastatin was related to different SLCO1B1 haplotype groups in 18 healthy Chinese subjects, with 6 in each haplotype group. This study showed that after baicalin administration, the systemic exposure of rosuvastatin significantly decreased compared to the placebo for SLCO1B1^*^1b/^*^1b (−41.9%) and SLCO1B1^*^1b/^*^15 subjects (−23.9%), but not for SLCO1B1^*^15/^*^15 subjects. A similar sample size of 20 subjects was used in the current study to explore the potential herb-drug interactions and their relationship with the ABCG2 transporter polymorphism.

## Results

### Establishment of Chemical Profiles of Herbal Products

#### Green Tea Extract Product

The green tea extract product mainly contained (–)-epigallocatechin-3-gallate (EGCG) as it corresponded with the EGCG standard on the chromatogram, although the other 6 chemical markers were also present in small amounts. The amount of EGCG within each sachet (daily dose) was 804.6, and the amounts of epigallocatechin (EGC), epicatechin (EC), epigallocatechin (EGC), gallic acid (GA), caffeine (CAF) and catechin (C) were 45.5, 5.9, 3.7, 1.02, 1.08, and 0.96 mg, respectively. The EGCG may have undergone some epimerization to gallocatechin gallate (GCG) during the extraction process which involved some heating and there was a small peak on the chromatogram close to the EGCG peak which was not identified or quantified, but may have been GCG.

#### Soy Isoflavone Product

The soy isoflavone product contained all 7 chemical markers, namely glycitin, daidzin, genistin, daidzein, glycitein, genistein and acetylgenistin in amounts of 58.64, 8.72, 6.48, 2.20, 4.23, 0.42, and 0.90 mg per sachet, respectively, and the total of these 7 soy isoflavones contents were calculated to be approximately 81.6 mg per sachet (daily dose). There were some other small unidentified peaks on the chromatogram which may represent other components of the extract that contribute to the total isoflavones.

### Effect of Green Tea Extract EGCG and Soy Isoflavones on the Pharmacokinetics of Rosuvastatin

**Figure 1:**
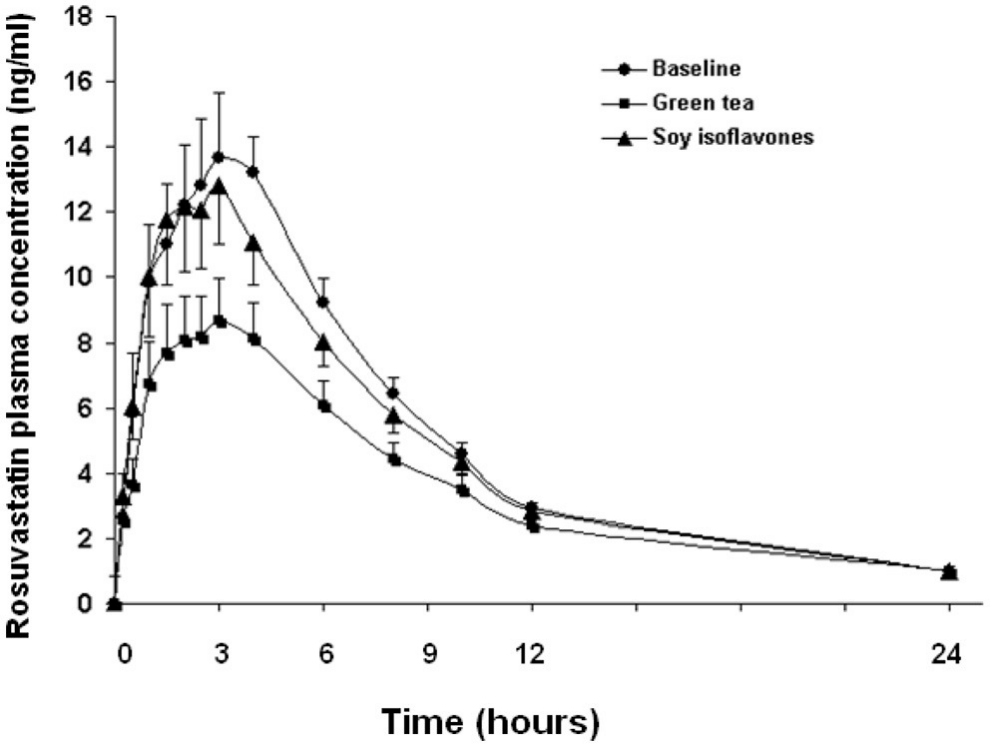
Effects of green tea extract and soy isoflavones on the pharmacokinetics of rosuvastatin in 20 healthy subjects. Data are arithmetic mean (±SD).

In the 20 healthy Chinese male volunteers [mean (±SD) age: 27.3 ± 5.8 years; body weight: 65.9 ± 7.0 kg; body mass index: 22.3 ± 1.9 kg/m^2^] (Table 1), intake of green tea extract (EGCG 800 mg daily for 14 days) significantly reduced the systemic exposure to rosuvastatin by 22% (geometric mean AUC_0−24*h*_ from 108.7 h·μg/L to 74.1 h·μg/L, geometric mean ratio 0.78, 90% CI 0.60–0.96, *P* < 0.001) and significantly reduced the C_max_ by 29% (geometric mean C_max_ from 13.1 to 7.9 μg/L, geometric mean ratio 0.71, 90% CI 0.53–0.89, *P* < 0.001) without affecting the elimination half-life (t_1/2_) (Figure 1; Table 2). Reduction in systemic exposure to rosuvastatin with EGCG was observed in 17 out of 20 subjects.

**Table 1:** Demographics of the 20 study subjects in the rosuvastatin-herb interaction study.

**Demographics**	**All subjects (*n* = 20)**	**ABCG2 421CC (*n* = 6)**	**ABCG2 421CA (*n* = 8)**	**ABCG2 421AA (*n* = 6)**
Age, years	27.3 ± 5.8	29.3 ± 5.1	23.5 ± 2.6	30.3 ± 7.3*
Body weight, kg	65.9 ± 7.0	63.8 ± 6.6	65.2 ± 7.5	69.0 ± 6.6
BMI, kg/m^2^	22.3 ± 1.9	21.9 ± 1.6	22.3 ± 2.4	22.6 ± 1.6

*p <0.05 vs. ABCG2 421CA.

**Table 2:** Effect of green tea extract and soy isoflavones on the pharmacokinetic parameters of rosuvastatin in 20 healthy subjects.

**Variable**	**Rosuvastatin**	**Rosuvastatin & green tea**	**Rosuvastatin & soy isoflavones**	**Overall P values**
C_max_, μg/L	13.1 (32.2)	7.9 (38.3)*	12.2 (39.1)	6 × 10^−6^
GMR (90%CI)	–	0.71 (0.53–0.89)	1.10 (0.82–1.37)	
AUC_0−24h_, h·μg/L	108.7 (28.9)	74.1 (35.3)*	101.0 (31.1)	<0.00003
GMR (90%CI)	–	0.78 (0.60–0.96)	1.03 (0.84–1.22)	
AUC_0−∞_, h·μg/L	117.5 (28.4)	84.0 (35.4)*	110.1 (30.0)	<0.00007
GMR (90%CI)	–	0.82 (0.63–1.00)	1.03 (0.85–1.21)	
Clearance, L/h	85.1 (28.4)	119.0 (35.4)	90.8 (30.0)	<0.00007
t_1/2_, h	6.21 (12.4)	6.99 (12.0)	6.50 (10.3)	0.225
t_max_, h	3.0 (1.5, 6.0)	3.0 (1.5, 6.0)	3.0 (1.5, 6.0)	0.748

*Data are geometric mean (% CV) except for t_max_ for which median (IQR) are given. GMR, geometric mean ratio compared to rosuvastatin alone value*.

*P <5 × 10^−5^ vs. baseline. AUC values in h·μg/L.

The soy isoflavones had no significant effect on the pharmacokinetics of rosuvastatin (Figure 1; Table 2).

### Effect of Genetic Polymorphisms on the Pharmacokinetics of Rosuvastatin and Its Interaction With Green Tea Extract

**Figure 2:**
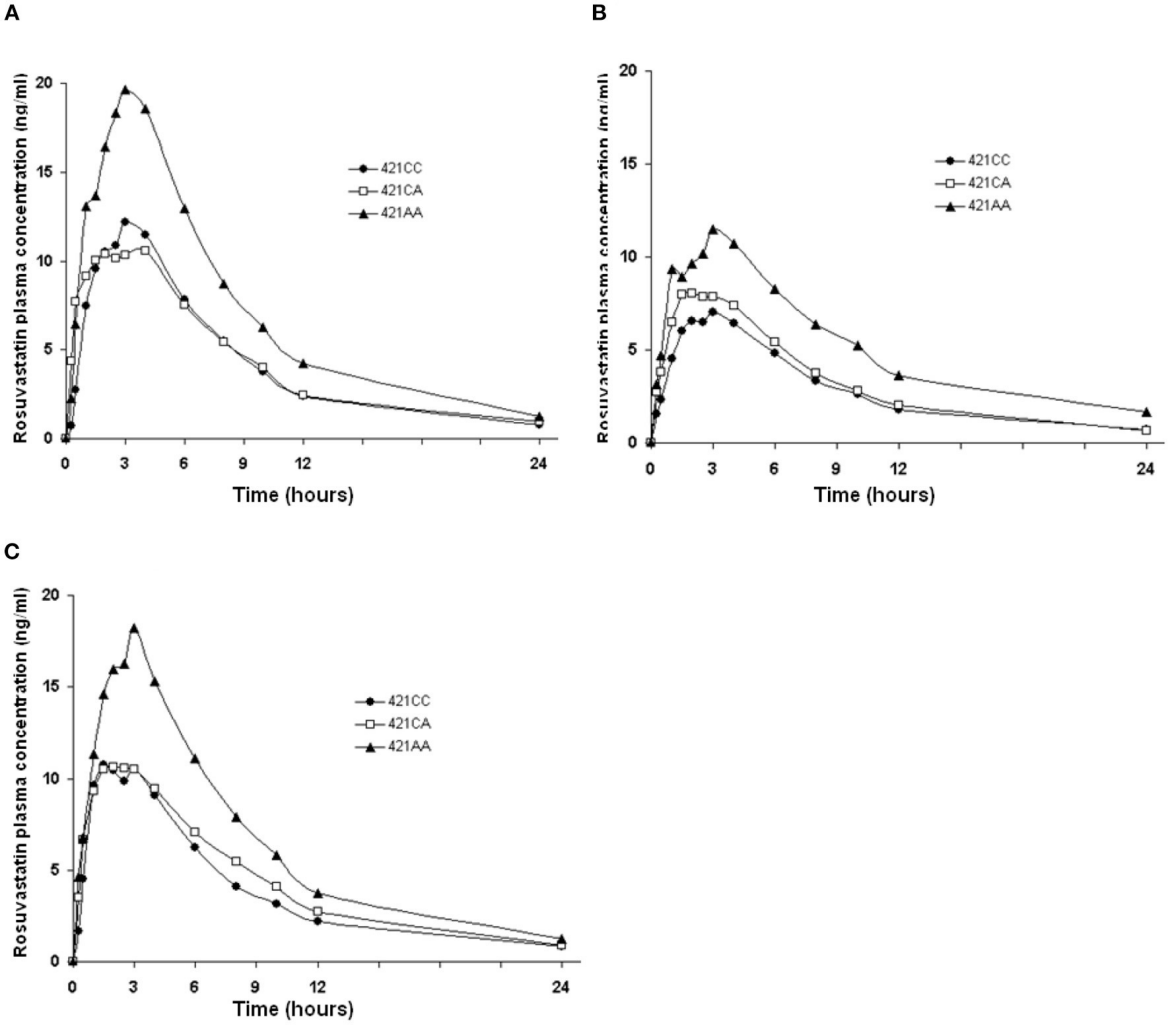
Effect of the ABCG2 421C>A polymorphism on the pharmacokinetics of rosuvastatin at baseline and after intake of green tea and soy isoflavones. Data are arithmetic mean. *P* < 0.05 for 421AA vs. 421CC + 421CA in all three phases. **(A)**, Baseline; **(B)**, After green tea extract; **(C)**, After soy isoflavones.

**Figure 3:**
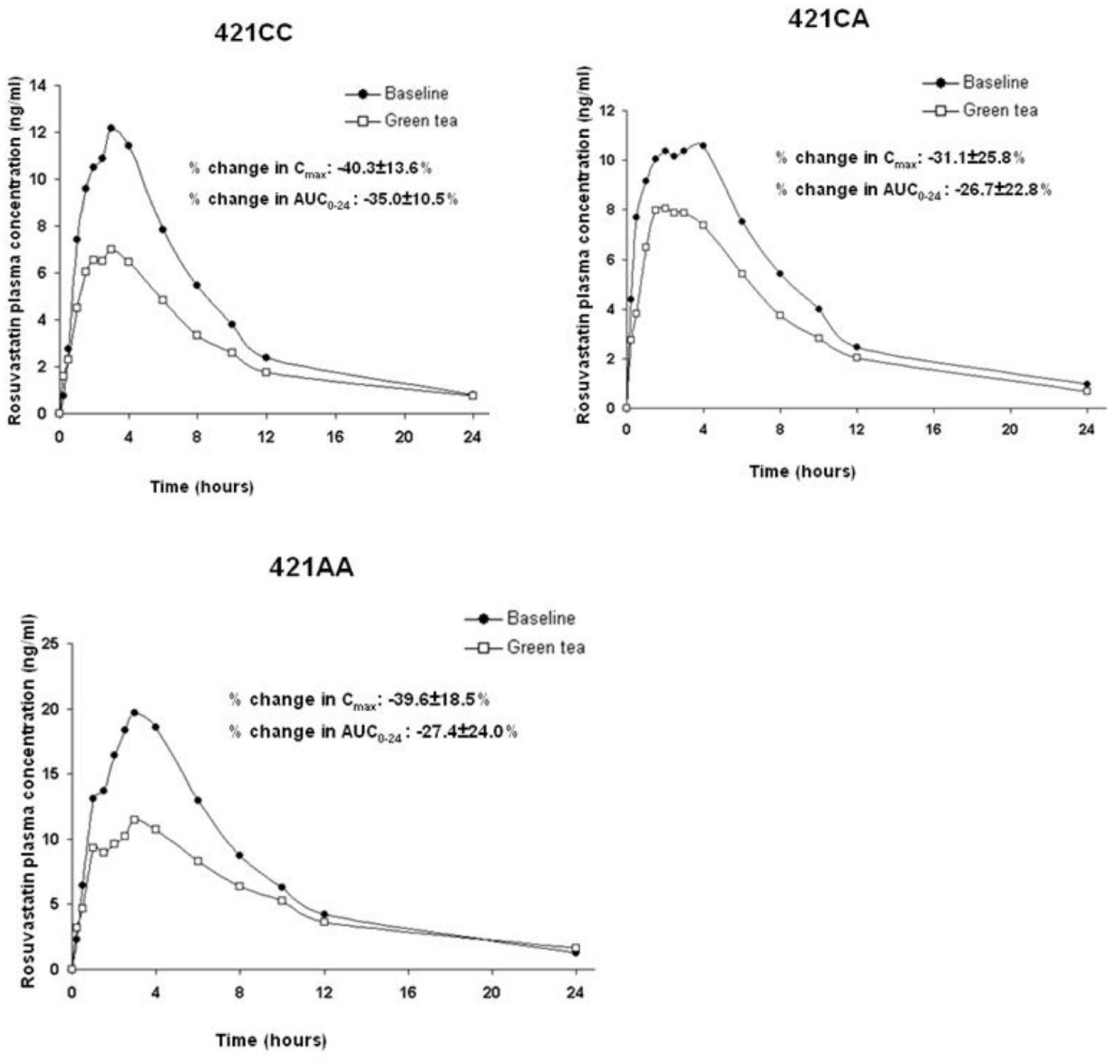
Effect of the ABCG2 421C>A polymorphism on the pharmacokinetic interaction between rosuvastatin and green tea. Data are arithmetic mean.

The ABCG2 421C>A polymorphism was associated with the pharmacokinetics of rosuvastatin in a recessive model. Subjects with the 421AA genotype had higher plasma concentrations of rosuvastatin than those with 421CC or 421CA genotype (*P* < 0.05) at baseline and after intake of green tea and soy isoflavones (Figure 2). However, the ABCG2 421C>A polymorphism had no significant effect on the interaction between rosuvastatin and the green tea extract (Figure 3).

### Adverse Events

The subjects did not report any adverse events during the periods of repeated intake of green tea extract or soy isoflavones or the single doses of rosuvastatin.

## Discussion

Beverages and food can interact with various medications and may alter their pharmacokinetic properties. In this study, we tested the hypothesis whether green tea or soy isoflavones would influence the pharmacokinetics of rosuvastatin, a commonly used statin, in Hong Kong Chinese subjects and whether genetic polymorphisms in drug transporters and drug metabolizing enzymes would affect the interactions. The most significant finding of the study is that intake of green tea extract with EGCG 800 mg daily for 14 days significantly reduced the systemic exposure of rosuvastatin by about 20% in healthy volunteers. The soy isoflavones had no significant effect on any of the pharmacokinetic parameters of rosuvastatin.

EGCG is the most abundant catechin in green tea, accounting for 50–80% of the total catechins and is a potent antioxidant that is believed to have the potential to treat a variety of human diseases such as cancer (30). Green tea or EGCG has also been reported to improve many cardiovascular risk factors, including plasma lipids, blood pressure, inflammatory biomarkers, oxidative stress, glucose and insulin resistance (31–33). The mechanisms by which green tea exerts its purported cardiovascular protective effects are subjects of interest in the field. Green tea catechin-enriched extracts are available over the counter as dietary supplements like vitamins and weight reduction pills and are widely and increasingly used by the general public, especially in those with increased cardiovascular risk, such as with hypercholesterolaemia. However, *in vitro* and *in vivo* studies suggested that green tea polyphenols may affect the absorption and metabolism of drugs by affecting the expression or activities of drug-metabolizing enzymes and drug transporters (34). It has been shown that green tea catechins inhibit the activities of CYP1A1, 1A2, 2A6, 2C9, 2E1, and 3A4 and may also induce the expression of CYP1A1, 1A2, 2D6, 2E1, 3A4 in cell lines (35, 36). Several studies have also indicated the inhibition of ABCB1 and ABCG2 activity by EGCG (37). Roth et al. showed that EGCG inhibited OATP1A2- and OATP2B1-mediated uptake of estrone-3-sulfate in a concentration-dependent manner in cells expressing these transporters (38).

On the other hand, EGCG was shown to activate OATP1B3 in *in vitro* studies (39). Various compounds can induce transporters such as P-gp and possibly hepatic OATP1B transporters by activating the pregnane X receptor (PXR) (40). The herbal medicine baicalin was shown to reduce the plasma concentrations of rosuvastatin in an OATP1B1 haplotype–dependent manner, suggesting the effect was mediated by induction of hepatic rosuvastatin uptake through OATP1B1 (29).

Another study reported that when rosuvastatin was administered with 300 mg of a pure crystalline formulation of EGCG, the AUC up to 8 h was reduced by 19% and C_max_ was reduced by 15%. However, after giving the EGCG daily for 10 days, the systemic exposure of rosuvastatin was not significantly different from the baseline value before EGCG, although the AUC and C_max_ values were intermediate between the values before and after a single dose of EGCG (41). The ABCG2 421C>A and SLCO2B1 935G>A (rs12422149) variants were associated with higher C_max_ and AUC values but did not affect the response to EGCG. The effect of the single dose was thought to be due to inhibition of intestinal uptake transporters OATP2B1 or OATP1A2, considering that the predicted plasma concentration would be sufficient to inhibit OATP2B1 (38). The authors speculated that long-term treatment might result in accumulation sufficient to inhibit liver uptake by OATP1B1 or to upregulation of enterocyte transporters. In addition to EGCG, other gallated catechins in green tea, such as ECG, can inhibit OATP1A2, OATP1B1, and OATP2B1 (38). Therefore, green tea may have a different effect from pure EGCG.

Rosuvastatin undergoes little metabolism, but it is a substrate of multiple transporters, including OATPs and ABCG2 (42). We and others previously showed that the loss-of-function mutation 421C>A in ABCG2 is a major genetic determinant of the pharmacokinetics and lipid-lowering effect of rosuvastatin (16, 43, 44). Similarly, inhibition of ABCG2 by green tea might also be predicted to result in increased plasma concentration of rosuvastatin. However, in this study in healthy volunteers, treatment with green tea extract containing mainly EGCG was associated with reduced systemic exposure to rosuvastatin. This may be explained by an inhibitory effect of EGCG on the intestinal uptake transporters OATP2B1 or OATP1A2, like the single-dose study of Kim et al. (45).

A study using cultured cells transfected with human P-gp or human BCRP showed no significant effect of green tea components EC, ECG, EGC, or EGCG on P-gp-mediated or BCRP-mediated dasatinib efflux, whereas some fruit juice components had a strong inhibitory effect (46). Lack of effect of the ABCG2 polymorphism on the effect of green tea extract on rosuvastatin pharmacokinetic in the present study would also suggest that the effect is not medicated by ABCG2 induction.

An animal study in male Sprague-Dawley rats showed that pre-treatment with green tea extract (400 mg/kg) as compared with control resulted in marked reductions in the C_max_ (by 85%) and AUC (by 74%) of nadolol, which is a substrate for several drug transporters and is not metabolized by CYP enzymes (47). EGCG alone (150 mg/kg) significantly reduced the C_max_ and AUC of nadolol by 81 and 73%, respectively (47). Green tea was shown to significantly decrease the C_max_ and AUC of nadolol by 85.3 and 85.0%, respectively, in 10 healthy volunteers (48). *In vitro* experiments revealed that nadolol is a substrate of OATP1A2 and green tea significantly inhibited OATP1A2-mediated nadolol uptake.

Further studies are needed to investigate the mechanisms underlying the interaction between rosuvastatin and EGCG. The most likely mechanisms appear to be inhibition of intestinal uptake by OAPT1A2 or activation of liver uptake by induction of OATP1B3 or OATP1B1. The former might be expected to reduce the lipid lowering effect of rosuvastatin whereas the latter might increase it.

### Limitations

This study has several limitations that need to be considered. Firstly, this study only assessed one dosage for each herb product (e.g., 800 mg EGCG and−80 mg soy isoflavones). It is known that interactions between herbs and drugs may be dose-dependent. Evaluating a higher dose or a lower dose may help to provide a better understanding of the interaction between statins and green tea or soy isoflavones. Secondly, it has been shown that taking EGCG 8 or 4 h before sunitinib administration had no effect on the pharmacokinetics of sunitinib in rats, whereas taking the two together reduced the bioavailability of sunitinib probably because of a physical reaction between the two compounds (49), suggesting separation of dosing of green tea and drugs may reduce any herb-drug interaction. To maximize the possibility of finding an interaction between statins and green tea, subjects were taking green tea extract and statins simultaneously on the dosing day, so that a physical interaction between the two substances cannot be excluded. It would be useful to assess whether the separation of dosing has different effects. During the study, we instructed the study participants to take the study product on an empty stomach (at least 1 h before breakfast after overnight fasting) to enhance the bioavailability of EGCG and to reduce the variations in EGCG bioavailability caused by food. They were requested not to take alcohol, tea, grapefruit juice, caffeine, soybean milk or dietary supplements and herbal products 2 weeks before and throughout the study. Subjects were educated about the importance of their compliance to the herbs and dietary restrictions for this research at the beginning of the study and they were reminded frequently of these requirements. The compliance to instructions on EGCG and soy isoflavones intake or dietary restrictions was monitored by using a food diary. This approach inevitably relied on the subjects' cooperation and honesty and may not be objective, but it was a practical way to conduct the study. It would be important to further evaluate the mechanisms responsible for the observed interactions and to assess whether these pharmacokinetic interactions have any impact on the lipid-lowering effect of statins in patients requiring long-term statin therapy.

## Conclusions

This study showed that repeated administration of green tea extract at a daily dose of 800 mg EGCG for 2 weeks was associated with decreased systemic exposure to rosuvastatin by about 20% in healthy volunteers when rosuvastatin was taken simultaneously with the green tea extract. There was no significant effect of soy isoflavones on rosuvastatin pharmacokinetics. Further studies should be performed to investigate the underlying mechanisms responsible for this observed interaction and to assess the clinical relevance in patients receiving long-term statins.

## Data Availability Statement

The original contributions presented in the study are included in the article/Supplementary Materials, further inquiries can be directed to the corresponding author/s.

## Ethics Statement

The studies involving human participants were reviewed and approved by the Joint Chinese University of Hong Kong-New Territories East Cluster Clinical Research Ethics Committee with reference number CRE-2010.524-T. The patients/participants provided their written informed consent to participate in this study. Written informed consent was obtained from the individual(s) for the publication of any potentially identifiable images or data included in this article.

## Author Contributions

WZ and MH analyzed the data and wrote this manuscript. BT designed the research project. HL, EW, CL, CW, and CH performed the experiments. BT and CH revised this manuscript. All authors contributed to the article and approved the submitted version.

## Funding

This study was supported by the Health and Health Services Research Fund of the Food and Health Bureau, Hong Kong Special Administrative Region Government (#09100321).

## Conflict of Interest

The authors declare that the research was conducted in the absence of any commercial or financial relationships that could be construed as a potential conflict of interest.

## Publisher's Note

All claims expressed in this article are solely those of the authors and do not necessarily represent those of their affiliated organizations, or those of the publisher, the editors and the reviewers. Any product that may be evaluated in this article, or claim that may be made by its manufacturer, is not guaranteed or endorsed by the publisher.
