# Supplementary material for: Effect of Green Tea Extract and Soy Isoflavones on the Pharmacokinetics of Rosuvastatin in Healthy Volunteers
Source: Front Nutr. 2022 Mar 24;9:850318. doi: 10.3389/fnut.2022.850318 (PMC8987933; doi:10.3389/fnut.2022.850318)
Supplement: Supplementary file 1 [file Data_Sheet_1.docx]

**Supplementary Material**

**Establishment of chemical profiles of herbal products**

The contents of the green tea extracts and soy extracts were verified in the Institute of Chinese Medicine laboratory, the Chinese University of Hong Kong using Ultra Performance Liquid Chromatography (UPLC) as below.

**Analysis of green tea extract**

UPLC analysis was performed using the ACQUITY UPLC® system from Waters Ltd., (Waters Ltd., Singapore), equipped with the ACQUITY UPLC® Sample Organizer, Column Manager, Binary Solvent Manager and Sample Manager (Waters Ltd., Singapore). Briefly, green tea extracts were injected onto a Supelco Discovery RP Amide C16 column (150 x 4.6 mm i.d., particle size 5 μm) (Sigma-Aldrich, Inc.). All solvents were pre-filtered with 0.45 μm Millipore filter disk (Millipore) and de-gassed. Gradient elution was carried out using the following solvent systems: mobile phase A - acetonitrile; mobile phase B - double distilled water/formic acid (99.9/0.1; v/v). The flow rate used was 0.8 ml/min and detection was performed at 280 nm. Each sample (5 μl) was injected into the column after filtration through a 0.45 μm filter disk. Identification of the tea polyphenols was carried out by comparing the retention times and the UV absorbance of the unknown peaks to those of the standards. A standard mixture containing 7 chemical markers: GA, EGC, C, EGCG, CAF, EC and ECG in methanol was prepared and analyzed. Calibration curves for EGCG, GA, EGC, C, CAF, EC and ECG were obtained using standard solutions injecting with volumes: 5, 10, and 20 μl. The system was monitored by a computer equipped with the Empower Software (Waters Ltd., Singapore) for data collection, integration and analysis.

**Analysis of soy extracts**

Soy extracts were analysed using the same UPLC and monitoring system from Waters Ltd., (Waters Ltd., Singapore) as for the green tea extract. Briefly, soy isoflavones extracts were injected onto a Supelco Discovery RP Amide C18 column (150 x 2.1 mm i.d., particle size 1.7 μm) (Sigma-Aldrich, Inc., USA). All solvents were pre-filtered with 0.45 μm Millipore filter disk (Millipore, USA) and de-gassed. Gradient elution was carried out using the following solvent systems: mobile phase A – acetonitrile/formic acid (99.9/0.1; v/v); mobile phase B - double distilled water/formic acid (99.9/0.1; v/v). The flow rate used was 0.4 ml/min and detection was performed at 254 nm. Each sample (5 μl) was injected into the column after filtration through a 0.45 μm filter disk. Identification of the soy isoflavones was carried out by comparing the retention times and the UV absorbance of the unknown peaks to those of the standards. A standard mixture containing 7 chemical markers (glycitin, daidzin, genistin, daidzein, glycitein, genistein and acetylgenistin) in methanol was prepared and analyzed. Calibration curves for all standards were obtained using standard solutions injecting with volumes: 5, 10, and 20 μl.
